# Identifying and Characterizing a Novel Protein Kinase STK35L1 and Deciphering Its Orthologs and Close-Homologs in Vertebrates

**DOI:** 10.1371/journal.pone.0006981

**Published:** 2009-09-16

**Authors:** Pankaj Goyal, Antje Behring, Abhishek Kumar, Wolfgang Siess

**Affiliations:** 1 Institute for Prevention of Cardiovascular Diseases, University of Munich, Munich, Germany; 2 University of Bielefeld, Bielefeld, Germany; Yale University, United States of America

## Abstract

**Background:**

The human kinome containing 478 eukaryotic protein kinases has over 100 uncharacterized kinases with unknown substrates and biological functions. The Ser/Thr kinase 35 (STK35, Clik1) is a member of the NKF 4 (*New Kinase Family 4* ***)*** in the kinome with unknown substrates and biological functions. Various high throughput studies indicate that *STK35* could be involved in various human diseases such as colorectal cancer and malaria.

**Methodology/Principal Findings:**

In this study, we found that the previously published coding sequence of the *STK35* gene is incomplete. The newly identified sequence of the *STK35* gene codes for a protein of 534 amino acids with a N-terminal elongation of 133 amino acids. It has been designated as STK35L (STK35 long). Since it is the first of further homologous kinases we termed it as STK35L1. The STK35L1 protein (58 kDa on SDS-PAGE), but not STK35 (44 kDa), was found to be expressed in all human cells studied (endothelial cells, HeLa, and HEK cells) and was down-regulated after silencing with specific siRNA. EGFP-STK35L1 was localized in the nucleus and the nucleolus. By combining syntenic and gene structure pattern data and homology searches, two further *STK35L1* homologs, *STK35L2* (previously known as *PDIK1L*) and *STK35L3*, were found. All these protein kinase homologs were conserved throughout the vertebrates. The *STK35L3* gene was specifically lost during placental mammalian evolution. Using comparative genomics, we have identified orthologous sets of these three protein kinases genes and their possible ancestor gene in two sea squirt genomes.

**Conclusions/Significance:**

We found the full-length coding sequence of the *STK35* gene and termed it as *STK35L1*. We identified a new third *STK35*-like gene, *STK35L3*, in vertebrates and a possible ancestor gene in sea squirt genome. This study will provide a comprehensive platform to explore the role of STK35L kinases in cell functions and human diseases.

## Introduction

Protein kinases mediate signal transduction in eukaryotic cells and are involved in regulating all cellular processes such as transcription, translation, cell cycle progression, cytoskeleton rearrangements, migration, apoptosis and differentiation. Eukaryotic protein kinases that phosphorylate serine/threonine (Ser/Thr) or tyrosine (Tyr) residues are the largest superfamily of enzymes, comprising ∼1.7% of all human genes [1]. Since completion of the first draft of the human genome sequence, various groups estimated the number of human kinases as 448, 510, and 518 depending on methods and data set used in the analysis [1]–[3]. Based on the current kinase databases (www.kinase.com; http://hodgkin.mbu.iisc.ernet.in/~king/), and a study by Stevan Hanks, the estimated number of human eukaryotic kinases (ePKs) is now 478 [4]. Most protein kinases have regulatory domains located C-terminal and/or N-terminal to the kinase domain. Despite the differences in their substrate specificities, the kinase domain of Ser/Thr- and Tyr- kinases is highly conserved and composed of two lobes with an overall length of roughly 275 amino acids [5]. The catalytic domain is characterized by a series of short sequence motifs, which define 11 sub domains and serve as key elements in the catalytic core of the kinase domain [6]. These motifs in combination with the overall catalytic domain sequence can be used to identify genes in genomes that encode protein kinases by various approaches such as sequence alignments and hidden Markov model searches. Such analysis and corresponding cDNA sequence information permits prediction of the number of protein kinases in different genomes [1]. The human kinome has over 100 uncharacterized kinases with unknown substrates and biological functions.

STK35 and its homolog PDIK1L (PDLIM1 (CLP36) Interacting Kinase 1 Like) shares 69% protein sequence identity in kinase domain. They are members of the NKF4 (New Kinase Family 4) Ser/Thr kinases (STK) family and classified in group “Other” in the human kinome [www.kinase.com; 1, 7, 8]. In Kinomer database, the best match for the STK35 kinase sequence is within the group TKL (Tyrosine Kinase Like) [9].

STK35 has also been named as Clik1 (CLP36 Interacting Kinase 1) based on one study that showed an association of STK35 with CLP36 after overexpression of both proteins in osteosarcoma cells [7]. CLP36 is an α-actinin binding protein, which contains a LIM- and a PDZ-domain and is mainly localized on stress fibers [10]. Therefore, a possible function of STK35 in the regulation of the actin cytoskeleton was proposed [7].

The biological function and substrates of STK35 are not known. The STK35 gene was found upregulated specifically in colorectum cancer [11]. The expression of STK35 gene was altered in a rodent model of Parkinson disease [12]. A kinome-wide RNAi screen revealed that STK35 silencing was among top five hits leading to reduced infection of hepatocytes by *Plasmodium berghei* sporozoites [13]. These studies suggest that STK35 may play a role in various human diseases deserving immediate attention by scientists working in different fields of biology and medicine.

In the present study, we describe the correct genomic organization and coding sequence of STK35, now renamed STK35L1, which was localized in the nucleus and nucleolus. We identified a new kinase subfamily containing three STK35L genes conserved in vertebrates. The present comprehensive study will provide a platform to further analyze the functional role and regulation of the STK35L kinases.

## Results

### EGFP-STK35 co-expressed with CLP36 does not translocate to stress fibers in endothelial cells

**Figure 1:**
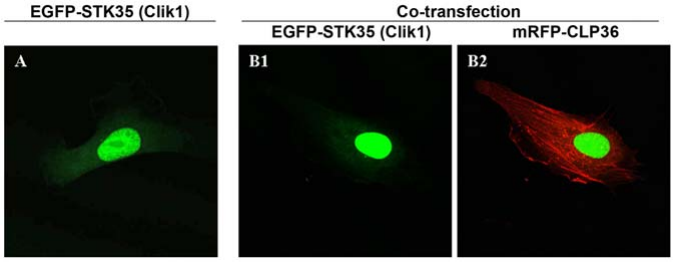
EGFP-STK35 (Clik1) does not translocate to actin stress fibers upon mRFP-CLP36 coexpression. Endothelial cells were transfected with EGFP-STK35 plasmid alone (A) or cotransfected with mRFP-CLP36 (B). The nuclear localization of EGFP-STK35 protein (A, green) was not affected by coexpression of mRFP-CLP36 (B1). mRFP-CLP36 (Red) was mainly localized on actin stress fibers (B2).

Previously, it has been shown that myc-tagged Clik1 (STK35) translocated from the nucleus to actin stress fibers upon coexpression with EGFP-CLP36 in U2OS osteosarcoma cells [7]. To test whether the translocation of STK35 to actin stress fibers could be observed in endothelial cells, we transfected endothelial cells with EGFP-STK35 plasmid alone or together with mRFP-CLP36 plasmid. In cells transfected with EGFP-STK35 only, we found the EGFP-STK35 protein predominantly nuclear with a faint cytoplasmic localization (Figure 1A). Unexpectedly, the coexpression of EGFP-STK35 and mRFP-CLP36 did not lead to translocation of STK35 to the cytoplasm and stress fibers (Figure 1B1 and 1B2) as reported previously. Moreover, we could not observe the presence of CLP36 in EGFP-STK35 immunoprecipitates from endothelial cells (data not shown). These data suggest that CLP36 interaction with STK35 does not occur ubiquitously. Therefore, the former designation of this kinase STK35 was used.

### Expression of STK35 RNA in various cell types

**Figure 2:**
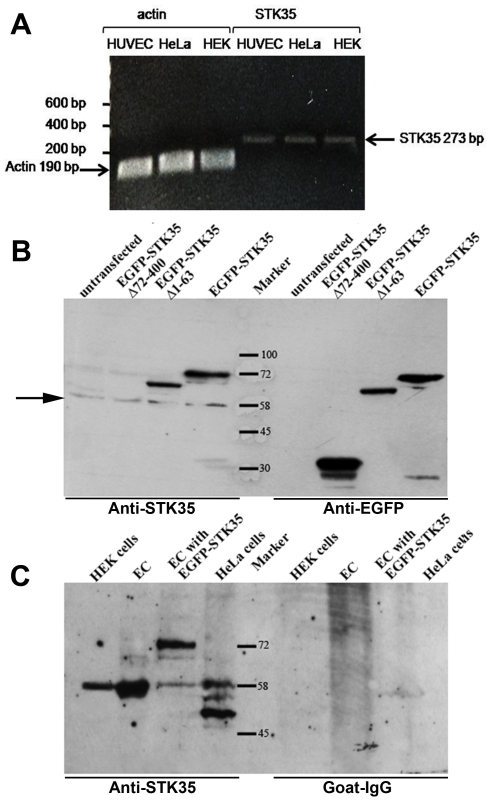
Expression analysis of STK35. (A) To verify the expression of STK35 mRNA, RNA and cDNA were prepared from endothelial, HEK293 and HeLa cells. 273 bp PCR products were amplified with STK35 specific primers from cDNA pools of endothelial, HEK and HeLa cells. β-actin amplification was used as a control. The primer pairs correspond to different exons to ensure the amplification from mature RNA. (B) Endothelial cells (EC) were transiently transfected with plasmids encoding different constructs of EGFP-STK35. Cell lysates of transfected or untransfected EC were immunoblotted with goat anti-STK35 antibody, or with mouse anti-EGFP as expression control. The detected bands correspond to the size of the fusion proteins (EGFP-STK35_Δ72-400: 35 kDa; EGFP-STK35_Δ1-6: 66 kDa; EGFP-STK35:72 kDa. The construct lacking the C-terminal part (EGFP-STK35_Δ72-400) was not recognized by anti.STK35 antibody. Note the additional band at 58 kDa in all lanes blotted with the anti-STK35-antibody (arrow). (C) Nuclei of EC, HEK and HeLa cells were isolated, and lysates were immunoblotted with goat anti-STK35 antibody or goat IgG as control. The bands detected by the antibody could not be found with the goat IgG control. In EC, HEK and HeLa nuclear lysates and in the whole cell lysate of EGFP-STK35 transfected cells a band of 58 kDa could be detected. In EGFP-STK35 (72 kDa) transfected cells, additional band of 72 kDa was detected. EC were transfected with pooled STK35L1 siRNA or control siRNA. After 24 to 72 hours of transfection, cell lysates were examined by western blotting with anti-STK35 antibody. After 72 hours, expression of 58 kDa band of STK35 protein was reduced by 80%. Actin blot was performed as control for equal loading.

To investigate STK35 mRNA expression in different cell types, total RNA was isolated from endothelial, HEK and HeLa cells and analyzed by RT-PCR. A fragment of the expected size for STK35 (273 bp) was amplified with STK35 specific primers (Table S1) in all cell types (Figure 2A). The correct sequence for STK35 fragment was confirmed by DNA-sequencing of the amplified product. Hence, endothelial, HeLa, and HEK cells express a transcript for STK35. By quantitative RT-PCR, we found that STK35 is equally expressed in these cell types (data not shown). To exclude a false positive amplification of possible DNA contamination of the isolated RNA, the primers for STK35 were designed to bind exactly at the exon-intron boundaries. No additional amplification of a genomic fragment of STK35 including the intron (13.3 kbp) was obtained in addition to the fragment derived from the STK35 mRNA transcript. In order to obtain further information about the expression profile of STK35 in human tissues and cell lines, we searched the Unigene database for Expressed Sequence Tags (ESTs) of STK35. 217 ESTs were found for the human STK35 gene, which are present in most of the tissues such as testis, ovary, skin, brain, heart, liver, and eye. To analyze the relative expression of STK35 RNA in various human tissues, we obtained the gene expression profile of STK35 from the SymAtlas database (http://symatlas.gnf.org) [14]. STK35 is expressed in all 79 human tissues studied. In testis and CD56+ NK cells, the expression was higher (Figure S1).

### Expression of STK35 in various cell types at protein level

To analyze the expression of endogenous STK35 protein in cells, rabbit polyclonal peptide antibodies against a C-terminal STK35 peptide were raised. To test the specificity of the antibody, cell lysates of untransfected and endothelial cells transiently transfected with plasmids encoding for EGFP-tagged STK35 proteins were used as negative and positive controls respectively. The antibody recognized the over-expressed proteins (∼72 kDa for EGFP-STK35) and a prominent band at about 58 kDa specifically whereas the pre-immune serum of the same rabbit did not detect these bands (data not shown). Importantly, we could not observe in transfected and non-transfected cells a band at 44.6 kDa, the theoretical calculated molecular weight of endogenous STK35. During the course of the study a commercial polyclonal goat anti-STK35 peptide antibody against the C-terminal amino acid sequence (ELETRMDQVTCAA) of STK35 became available. This antibody recognized the EGFP-STK35 fusion proteins which contain the C-terminal part of the protein specifically (EGFP-STK35, EGFP-STK35Δ1-63) but not a truncated construct lacking this sequence (EGFP-STK35Δ72-400) (Figure 2B). The blot incubated with mouse anti-EGFP antibody clearly showed a band of this truncated construct confirming its expression in cells (Figure 2B). Beside the detection of the EGFP-tagged STK35 proteins, again a band of 58 kDa was specifically recognized by the goat anti-STK35 antibody but not by the anti-EGFP antibody (Figure 2B). This indicates that this 58 kDa band could represent endogenous STK35 protein.

To analyze further, nuclear lysates of endothelial, HEK and HeLa cells were prepared. A prominent band at 58 kDa was recognized by the anti-STK35 antibody in nuclear extracts of HEK, HeLa and endothelial cell (Figure 2C) and macrophages (data not shown). In HeLa cells two additional bands at about 52 kDa and 48 kDa were detected. The control blot probed with goat IgG did not show any bands. The theoretical molecular weight of STK35 (44.6 kDa) is significantly less than the molecular weight of the endogenous protein (58 kDa) recognized by these two different antibodies. Posttranslational modification as explanation for such a large mobility shift from 45 kDa to 58 kDa seemed to us unlikely. Moreover, overexpressed EGFP-tagged STK35 would be expected to undergo the same posttranslational modifications as endogenous STK35 in endothelial cells but this was not the case: the detected molecular weight of this tagged protein corresponded exactly to its calculated molecular weight. We also examined the possibilities of longer *STK35* gene splice variants from data set of Ensembl and NCBI human build. However, no splice variants co-relation can be established. Therefore, we undertook a careful genome analysis of the STK35 locus.

### Identification of STK35 protein coding region in different genomes

**Figure 3:**
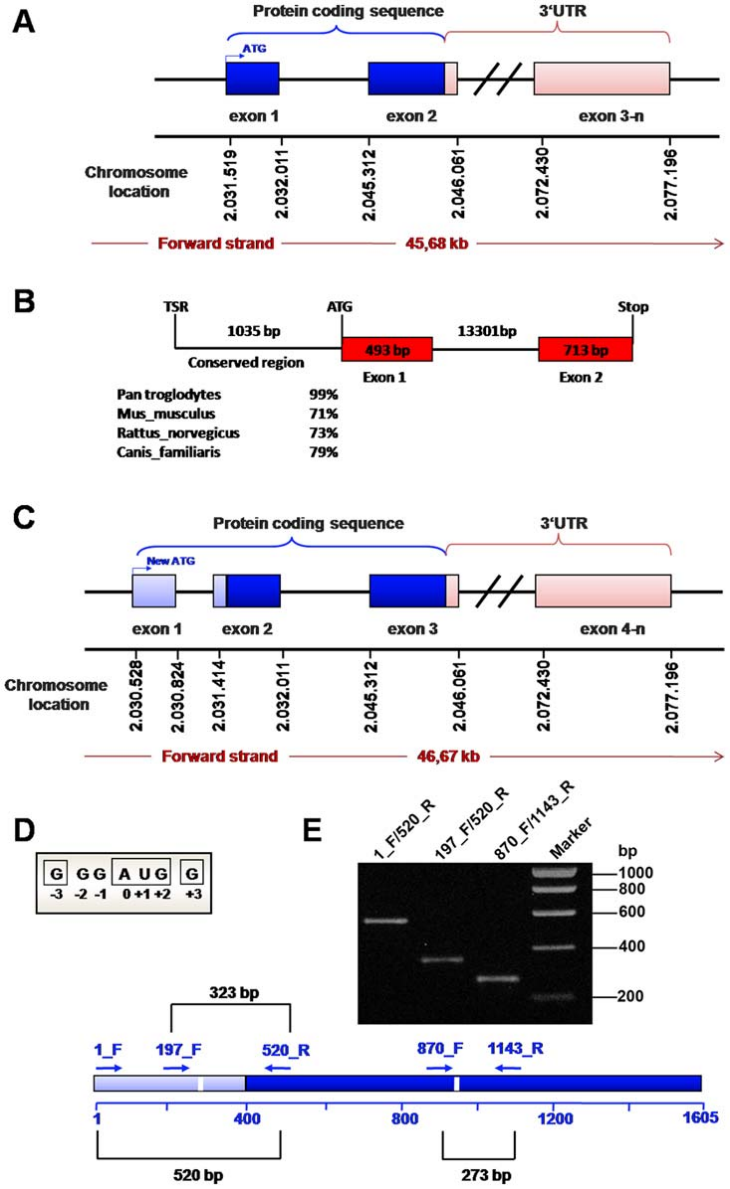
Genomic analysis of STK35L1. (A) Schematic representation of the published STK35 (Clik1) gene. The location on chromosome 20 is shown. The coding region is shown in blue. (B) The structure of the STK35 locus after identification of the putative TSR. The 1035 bp sequence between TSR and the start codon of the published gene is highly conserved among mammals as shown in % identity. (C) Schematic representation of the *STK35L1*gene. The 5′ extended sequence is marked in light blue. (D) The putative start codon of the *STK35L1* gene matches the Kozak consensus sequence RccAUGG. Some nucleotides in this sequence are more important than others. For a ‘strong’ consensus, the nucleotides at positions +3 (G) and −3 (R = A or G) must both match the consensus. [42]. E) mRNA expression of the *STK35L1* gene. Upper panel: Amplification of the expressed transcripts of *STK35L1* from human EC cDNA. The lower axis: the putative mRNA transcript of *STK35L1* gene and its length in base pairs. The extended sequence to the 5′ end is shown in light blue, the previously published sequence is represented in dark blue. Positions of introns are indicated as white bars. Arrows over the axis represent the relative position of primers used for the amplification of cDNA. The sizes of the three obtained PCR products are indicated.

**Figure 4:**
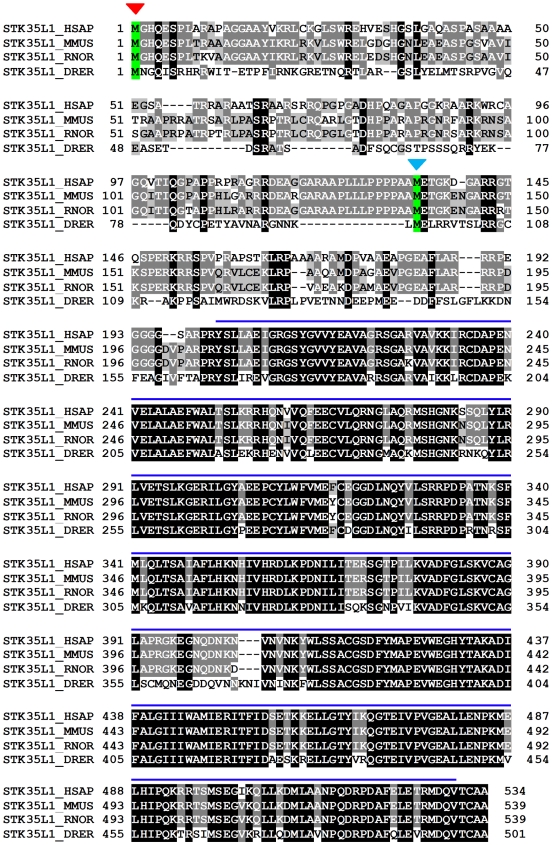
Alignment of protein sequence of STK35L1 from different vertebrates human (HSAP), mouse (MMUS) rat (RNOR), and zebrafish (DRER). Red arrow indicates the newly identified start codon while blue arrow head indicates start codon previously published. The position of kinase domain is indicated by blue line. The STK35L1 kinase domain of zebrafish has 81% identity with human kinase domain.

To identify the open reading frame of the STK35 gene, we analyzed the genomic sequence of the STK35 locus in different genomes from different representative species of vertebrate phyla using various gene structure prediction tools. First we used the ElDorado tool of Genomatix GmbH (http://www.genomatix.de/). We used STK35 cDNA (NM_080836) sequence for complete analysis of the STK35 locus in human genome. The STK35 gene is located on chromosome 20 subregion p13 (Figure 3A). The chromosomal region NC_000020 between 2.013.772 and 2.063.771 (50.000 bp) was analyzed. Three transcription start regions (TSR_2.030.439–484, TSR_2.030.648–690 and TSR_2.031.306–335) were found based on CAGE (Cap-analysis gene expression) tags profile. CAGE is a high-throughput method to measure expression levels of a gene and is an essential resource for profiling transcriptional starting sites [15], [16]. The transcription start region TSR_2.030.439–484 was 1035 bp upstream of the previously defined STK35 start codon. This region was highly conserved among mammals (Figure 3B). We investigated this region for coding sequences manually and by using different gene prediction software such as FGENESH (www.softberry.com). We identified a start codon surrounded by a Kozak consensus sequence and an open reading frame encoding a protein of 534 amino acids instead of the 401 amino acids predicted earlier for STK35 (Figure 3C and 3D). The theoretical molecular weight of the predicted protein was 58 kDa which matched completely the molecular weight of the endogenous protein on the immunoblots. Furthermore, we found that the new STK35 gene has 3 coding exons (Figure 3C). The newly identified STK35 gene structure is maintained from fish to mammals (Figure 4). In contrast, the previously identified protein start codon of the STK35 gene is found in the middle of second exon.

To investigate the presence of mRNA transcripts of the potentially longer form of STK35 experimentally, endothelial cell total RNA was isolated and analyzed by RT-PCR. The primers were designed to span the introns boundaries and to combine the extended predicted sequence with the published sequence of STK35 (Figure 3E). The RT-PCR results show an expression of mRNA transcripts of the newly identified STK35 gene including exon 1 (Figure 3E). The correct sequences of these PCR-amplified products were confirmed by DNA-sequencing (data not shown). Possible genomic DNA contamination was excluded by using negative control (reverse transcriptase polymerase was excluded during RT-PCR) of RT-PCR samples (Figure S3). This data indicates that the longer form of STK35 is expressed in mammals. Hence, based on our finding we termed the full length gene of STK35 as STK35L (STK35 Long). Since it represents the first member of NKF4 family, it is named STK35L1.

### Knockdown of endogenous STK35L1 expression by siRNA

**Figure 5:**
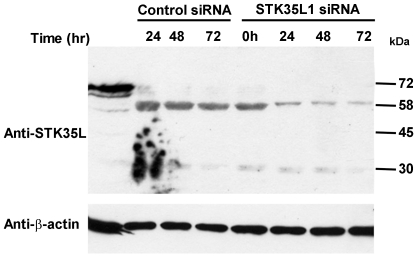
Knockdown of STK35L1 protein expression. To knockdown the STK35L1 protein expression, EC were transfected with pooled STK35L1 siRNA or control siRNA. After 24 to 72 hours of transfection, cell lysates were examined by western blotting with anti-STK35 antibody. After 72 hours, expression of 58 kDa band of STK35 protein was reduced by 80%. Actin blot was performed as control for equal loading.

In order to prove that the endogenous 58 kDa protein band is STK35L1, we used the siRNA approach to knockdown the expression of endogenous STK35L1. We designed siRNA directed against three different regions of STK35L1 gene (Table S1). Indeed, the expression of 58 kDa band was highly reduced in endothelial cells (by ∼80%) after transfecting a pool of the three siRNA oligonucleotides (Figure 5). One siRNA oligonucleotide (siSTK35L_1111) could downregulate 70% of the 58 kDa protein, whereas the others (siSTK35L_839 and siNSTK35L_5816) were less effective (data not shown). These data confirm that the endogenous 58 kDa protein is STK35L1.

### Subcellular distribution of STK35L1

**Figure 6:**
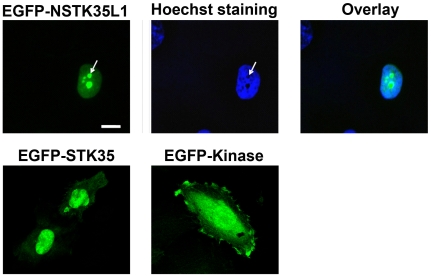
Subcellular distribution of STK35L1 and its mutants in endothelial cells. Cells transfected with EGFP-STK35L1 or deletion mutants were fixed and DNA was stained with Hoechst dye. EGFP-NSTK35L1 is mainly localized in nucleus and nucleolus (left panel white arrow). The nucleolus is excluded by Hoechst-dye staining (middle panel white arrow). In contrast, EGFP-STK35 is mainly localized in nucleus. EGFP-Kinase (N-terminal 1–196 amino acids of STK35L1 were deleted) is diffusely distributed in the nucleus and the cytoplasm indicating that N-terminal part of STK35L1 has functional nucleolar localization signal.

To analyze the subcellular distribution of STK35L1, EGFP-STK35L1 plasmids were transfected into endothelial cells and analyzed by fluorescent microscopy. The protein accumulated primarily in the nucleus. Moreover, EGFP-STK35L1 was concentrated in dense bodies in the nucleus (Figure 6, white arrow), that were easily identified as nucleoli by phase contrast microscopy and exclusion of Hoechst DNA staining dye (Figure 6). The construct deleted of the first 196 N-terminal amino acids (EGFP-Kinase), containing only the kinase domain, diffusely distributed throughout the cytoplasm and the nucleus (Figure 6). These data suggest that the N-terminus of STK35L has a functional nuclear and nucleolar localization signal.

### Identification of *STK35L1* orthologous set and gene conservation in vertebrates

**Figure 7:**
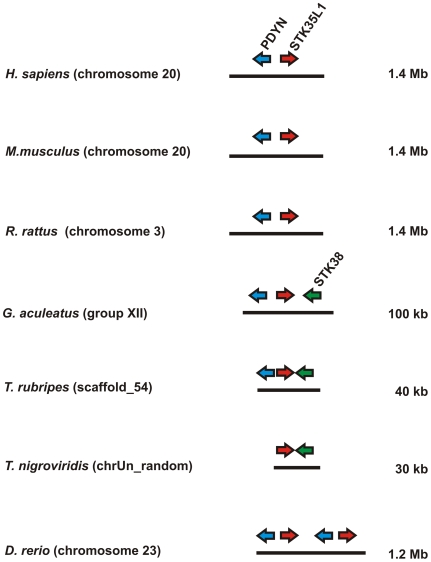
Genomic localizations of *STK35L1* and flanking genes in different vertebrates.

Gene synteny is a useful tool for predicting functional interaction and relatedness among genes within cluster. Selective processes are essential to preserve the organization of these clusters in closely related species. In the human genome, the *STK35L1* gene is localized on chromosome 20. On further inspecting this gene in different vertebrates genome (Table S2), we found that this gene is maintained in fish, *Xenopus* and in all mammals. To ensure orthology of this gene, syntenies amongst different vertebrates were created. From human to fish, the *STK35L1* gene is flanked by the marker gene *PYDN* (prodynorphin) in an inverse manner (Figure 7). Moreover, the *STK35L1-PYDN* region has tandem duplication resulting in two copies of these genes in the *Danio* genome. These data infer that flanking marker genes are conserved in micro-syntenic environment corresponding to *STK35L1* synteny. *STK35L1* orthologs and species-specific paralogs are listed in Table S3. Interestingly, the *PDYN- STK35L1* locus is not found in *Gallus* genome using *PDYN* as marker, since *PDYN* was not detectable in the same genomic locus. However, we could identify the *STK35L1* locus by using some other conserved markers (data not shown). By homology search in *Gallus* genome, we found that the *STK35L1* gene was truncated and the exon 1 and 2 were missing. The expression of the truncated *STK35L1* gene in *Gallus* is supported by an mRNA sequence submitted in the gene bank (BU403355).

### Identification of STK35L2 orthologous set in vertebrates

**Figure 8:**
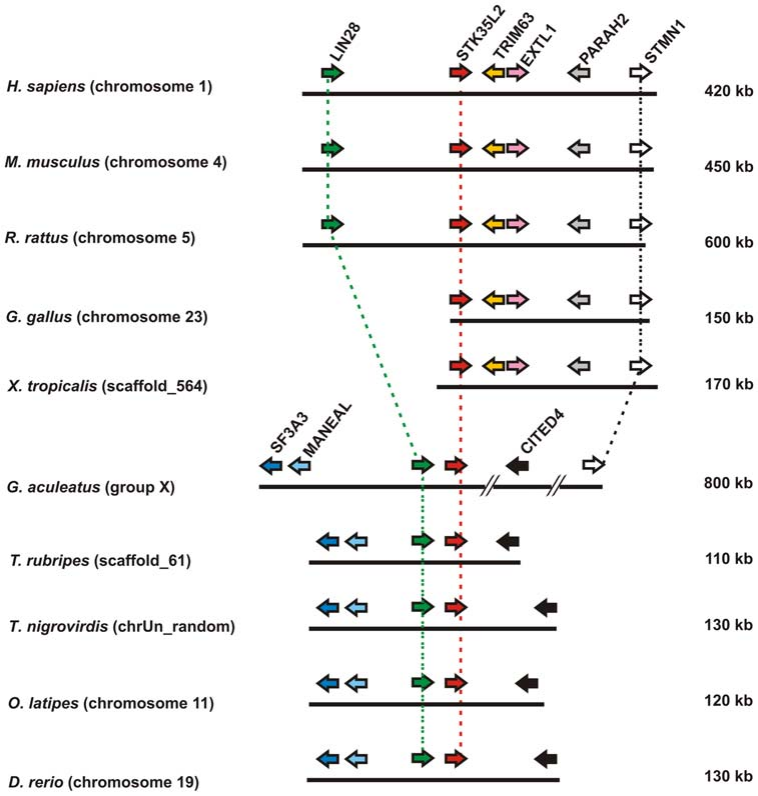
Genomic localizations of *STK35L2* and flanking genes in different vertebrates.

*STK35L2* (previously known as *PDIK1L*) is localized on chromosome 1 in human. In mammals, *STK35L2* was conserved differently: the locus is flanked by a marker tetrad *EXTL1-TRIM63-PHARAH2-STMN1* (Table S4 for markers details) on one side and the marker gene *LIN28* on the other side (Figure 8). However, in frog and chicken genomes, this gene was only flanked by tetrad *EXTL1-TRIM63-PHARAH2-STMN1* on one side. In fish, the marker sets were not totally conserved: on one side, the CITED4 gene was present, on the other side, two additional flanking genes–*MANEAL and SF3A3* (Table S4) were present in inverse orientation to the conserved *LIN28. STMN1* gene is maintained from human to *G. asculeatus* within an 800 kb fragment. Jointly, *LIN28* and *STMN1* aided in deciding orthology of *STK35L2* gene locus. Hence, this suggests that *STK35L2* has an independent origin from *STK35L1*. The human orthologs of *STK35L1*and *STK35L2* were maintained from fish to humans. *STK35L2* orthologs and species-specific paralogs are listed in Table S3.

### Identification of the novel homolog *STK35L3* in vertebrates and characterizing its orthologous set in different vertebrates

During investigation of *STK35L1* and *STK35L2* genes from different vertebrates, we found that some vertebrates possess a novel *STK35L* like gene. This gene is not reported in the literature by today. Since two homologs of the *STK35L1* gene are already known, we named this gene as *STK35L3*. Astonishingly, this gene is maintained from fish to mammals as listed in Table 1 with accession ids and genomic location.

**Table 1:** Orthologous set of *STK35L3* from different vertebrates.

Organism	Species	Accession id#	Genomic location
**Opossum**	*Monodelphis domestica* (Mdom)	ENSMODG00000008648	Chromosome 1: 589,412,721-589,435,590
**Chicken**	*Gallus gallus* (Ggal)	AJ720485.1*	Chromosome 3: 108,470,800-108,479,700
**Frog**	*Xenopus tropicalis* (Xtra)	ENSXETG00000021156	Scaffold_470: 254,754-259,637
**Zebrafish**	*Danio rerio* (Drer)	ENSDARG00000042000	Chromosome 20: 32,550,616-32,559,734
**Pufferfish**	*Takifugu rubripes* (Trub)	ENSTRUG00000002570	Scaffold_143: 136,478-140,245
**Green spotted pufferfish**	*Tetraodon nigroviridis* (Tnig)	ENSTNIG00000019170	Chromosome 14: 1,220,644-1,222,657
**Stickleback**	*Gasterosteus aculeatus* (Gacu)	ENSGACG00000004453	GroupXVIII: 1,071,040-1,077,095
**Medaka**	*Oryzias latipes* (Olat)	ENSORLG00000016204	Chromosome 24: 13,431,187-13,437,480

#–Ensemble accession id.

* –NCBI accession id.

**Figure 9:**
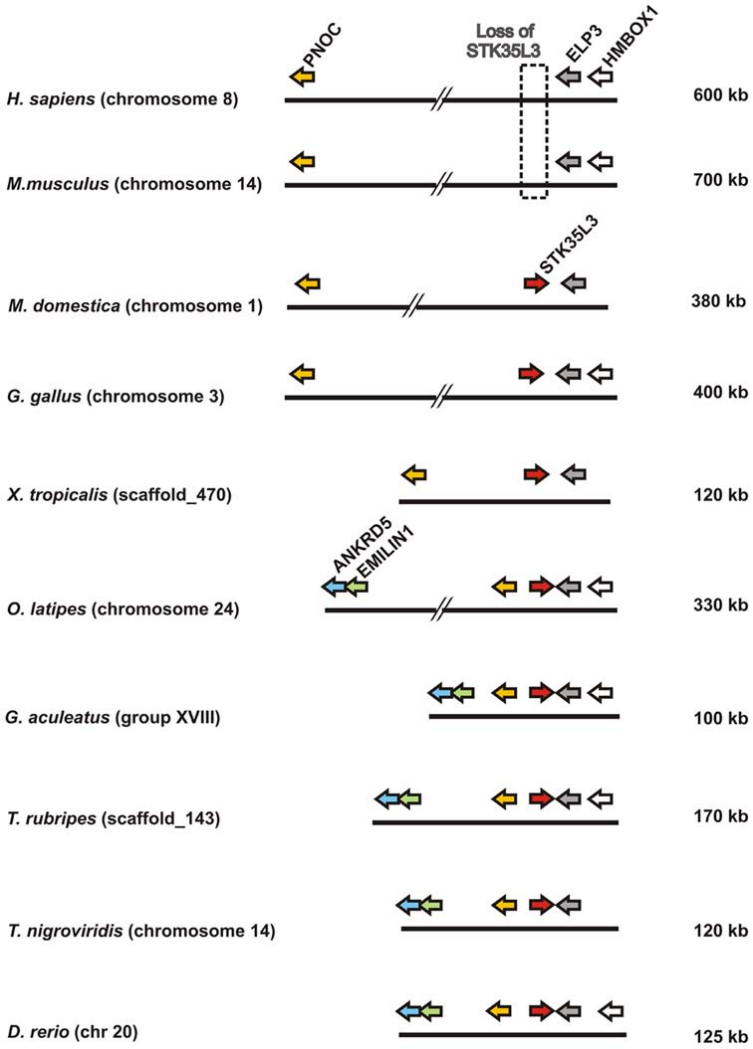
Genomic localizations of *STK35L3* and flanking genes in different vertebrates.

To unravel the origin of *STK35L3* gene, the micro-genomic organization flanking the *STK35L3* gene was analyzed (Figure 9). In *M. domestica* (opossum) genome, *STK35L3* is gene flanked by *PNOC* and *ELP3* (Table S4 for markers details) in a ∼380 kb region on chromosome 1. A similar locus is conserved in a ∼120 kb fragment on scaffold_470 in frog genome. However in fish genomes, this locus contains two additional conserved markers *ANKRD5* and *EMILIN1* (Table S4) flanking in inverse orientation. Conservation of locus of *STK35L3* suggest that the *STK35L3* gene is found in orthologous condition from fish to mammals, and that the *STK35L3* does not result by tandem duplication of *STK35L* genes as they are found in distinct syntenic organization. On further investigating this gene in different mammals, we found that this gene is lost in eutherian (“placental”) mammals (such as human and mouse in Figure 9), whereas it is retained in metatherian (“marsupial”) mammals (as in opossum).This suggests that *STK35L3* gene in eutherians is lost after metatherian\eutherian divergence ∼173–190 MY ago [17], [18], [19]. Hence, we corroborate the finding of novel *STK35L3* ortholog in marsupial mammals, chicken, frog and at least in five fish genomes.

### Evolution of STK35L1 and its homologs

**Figure 10:**
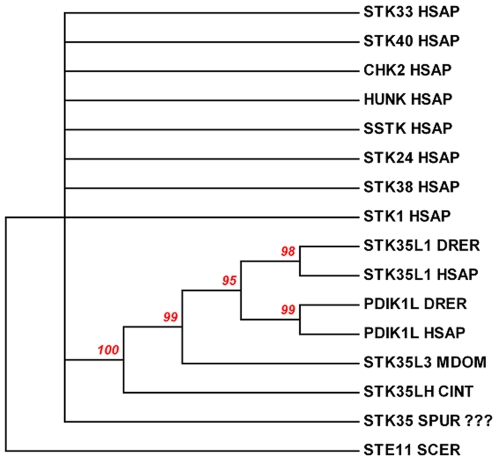
Evolutionary relationships of STK35L1 and its homologs with selected kinases. The previously published [24] probable STK35 gene of Sea urchin is not STK35 gene as do not cluster with STK35 like genes from other genomes with 60% bootstrap values. In contrast, *Ciona* STK35 gene is clustered with STK35 like genes. The evolutionary history of selected kinases was inferred using the Neighbor-Joining method [26]. The bootstrap consensus tree inferred from 1000 replicates is taken to represent the evolutionary history of these selected kinases [25]. Branches corresponding to partitions reproduced in less than 60% bootstrap replicates are collapsed. The percentage of replicate trees in which the associated kinases clustered together in the bootstrap test (1,000 replicates) are shown above the branches. The evolutionary distances were computed using the Poisson correction method and are in the units of the number of amino acid substitutions per site. All positions containing gaps and missing data were eliminated from the dataset (Complete deletion option). There were a total of 227 positions in the final dataset. Phylogenetic analyses were conducted in MEGA4. Outgroup is yeast STE11 gene choose from www.kinase.com. SCER–yeast, HSAP–human, DRER- zebrafish, MDOM–opossum, CINT–sea squirt, SPUR–sea urchin.

Our data clearly demonstrated that there are distinct orthologous sets of STK35L1 and its homologs in different vertebrate species. Since these are maintained from fish to human, this indicates that an ancestor gene might be present before the emergence of vertebrates or that these genes arose at the point of vertebrate origin. To identify an ancestor gene of *STK35L1*, homology searches in different invertebrate genomes were carried out. By searching invertebrate model genomes (Table S2) such as *Branchiostoma floridea* (lancelets) [20], *Strongylocentrotus purpuratus* (sea urchins) [21], *Drosophila melanogaster* (flies) [22], and *Caenorhabditis elegans* (worms) [23], no genuine homologs were found, although these species possess many eukaryotic protein kinases. However, we identified a *STK35L1* like gene in two genomes of sea squirts - *Ciona intestinalis* (JGI Ciona genome accession id -fgenesh3_pg.C_chr_07q001099/Ensembl - ENSCINP00000006268) and *Ciona savignyi* (Ensembl–ENSCSAV00000013846) with identity of ∼45% with human *STK35L1* (Figure S2). Moreover, in a previous study on the sea urchin kinome [24], *STK35* kinase gene (SPU_003710) had been reported. However by careful comparison of the protein sequence of sea urchin kinase with the STK35L1 protein, we found that the sea urchin kinase shared only 10% identity with STK35L1 in the kinase domain. To confirm whether the sea urchin kinase is clustered together with STK35L1 homologs or not in the evolutionary tree, we created a phylogenetic tree of randomly selected representative kinases with STK35 homologs, sea urchin and *Ciona* STK35L1 like kinases (Figure 10) by using neighboring joining method [25], [26]. The status of the sea urchin kinase being STK35 is highly questionable as it did not cluster along with STK35L1 homologs in this phylogenetic tree with a statically significant value (the bootstrap value is below 60%). This sea urchin kinase randomly located as other representative kinases in this tree. In contrast the *STK35* like gene in *Ciona* species is grouped with STK35L1 homologs with a bootstrap value of 100%. These data suggest that *Ciona* kinase but not sea urchin kinase is the closest homlog of the vertebrate STK35L1.

**Figure 11:**
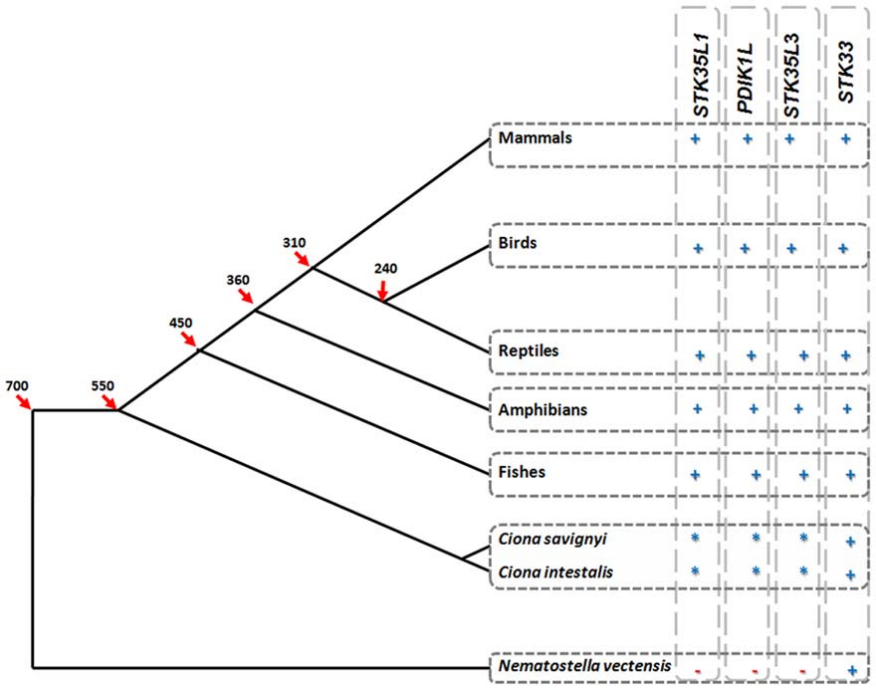
Evolution of *STK35L1* homologs in different metazoan genomes. These genes most probably originated from a *STK35L1* homolog from sea squirt. This finding dates the origin of *STK35L1* homologs to 550 MY ago. The *STK33* gene (28% identity with *STK35L1* in the kinase domain) is found already in the sea anemone genome (accession id e_gw.81.82.1), dating ∼700 MY ago. The estimated divergence times (in MY) were taken from [19] and are marked with red arrows. +, presence, -, absence, *, ancestor gene. ?, information not available.

To compare the evolution of *STK35L1, STK35L2, STK35L3* on a time scale, a time-wise gene distribution of these STK35L1 homologs was created (Figure 11). This finding indicates that STK35L1 homologs were present from 550 MY ago with separation of sea squirt lineage from vertebrate lineage. Notably, the *STK33* is the closest homolog of the *STK35L1*gene (28% identity of the kinase domain) in comparison to other kinases. An ancestor gene of STK33 is found in sea anemone genome [27] from the beginning of metazoan dating ∼700 MY ago.

### Gene structures of STK35L1 homologs

**Figure 12:**
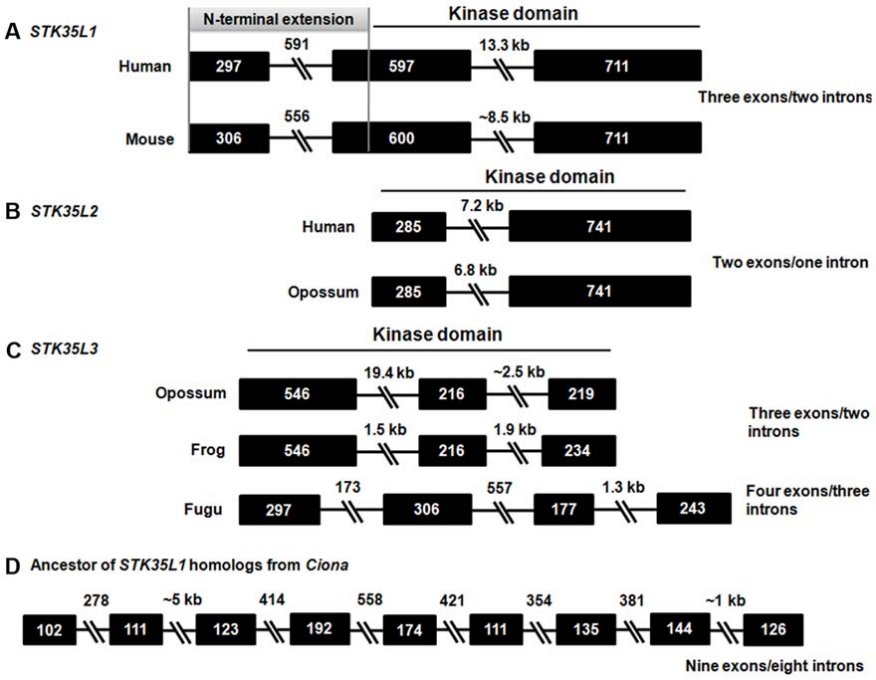
Variable exon-intron organization of *STK35L1* homologs and their ancestor gene from *Ciona*. Exons are depicted by boxes and introns by broken lines and labeled with their indicated sizes (bp). An extension of 603 bp of STK35 gene comprising of full exon 1 and part of exon 2 codes the N-terminal extension of the protein (marked in grey color). Kinase domains are indicated by black line.

Gene structures attribute phylogenetic evidence to genes and aid in assigning origin of given genes. To compare gene structures, exon-introns architectures of different *STK35L1* homologs were determined. *STK35L1* homologs have variable exon-intron organization patterns (Figure 12). *STK35L1* and *STK35L2* genes in different vertebrates possess gene structures with ‘three exons/two introns’ (Figure 12A) and ‘two exons/one intron’ (Figure 12B) patterns, respectively. *STK35L3* from marsupials, chicken and frog possess a ‘three exons/two introns’ (Figure 12C) pattern. However *STK35L3* from fish carries a discernable ‘four exons/three introns’ gene structure pattern, indicating intron loss from fish to human. A nine exons/eight introns gene structure pattern is found in ancestor gene of *STK35L1* from *Ciona* (Figure 12D), suggesting that the vertebrate *STK35L1* gene arose by intron losses event at the point of vertebrate emergence. The analysis of exon-intron structures has proved to be a valuable method for determining phylogenic relatedness of *STK35L1* homologs as it suggests independent origin of these orthologous sets of *STK35L1* homologs. Otherwise, a conservation of the intron–exon organization among these three types of genes would have been maintained. Additionally, the gene structure patterns of *STK35L1* homologs do not resemble that of the closest homologs of serine threonine kinases (such as *STK33/STK38/STK40*). This corroborates that *STK35L1* homologs do not share orthology with other serine threonine kinases.

## Discussion

In the present study we found that the previously published coding sequence of the *STK35* gene (code for 401 amino acids) is incomplete. The newly identified sequence of *STK35* gene codes for a protein of 534 amino acids which was termed as STK35L1. By western blot analysis of endothelial cell lysates using two different specific polyclonal anti-STK35 antibodies, we found that STK35L1 was expressed in all mammalian cells studied. By using the siRNA approach, we could confirm that the 58 kDa protein is endogenously expressed STK35L1 protein. In contrast, no evidence for the expression of STK35 was obtained by western blotting using the antibodies directed against the C-terminal peptide of STK35.

Endogenous STK35L1 was found to be localized in the nucleus. Moreover, EGFP-STK35L1 was also localized in the nucleolus suggesting a role in gene regulation. A previous report suggested that STK35 may have role in actin cytoskeleton rearrangement as the interaction of the co-transfected STK35 and CLP36 proteins led to relocalization of Clik1 from the nucleus to actin stress fibers [3]. In endothelial cells, this result could not be confirmed. Recent studies suggest that STK35 could play a role in various diseases [11], [12], [13].

The identification of the full protein coding gene sequence is obviously crucial for the determination of the biological function of the kinase. Therefore, we analyzed the *STK35* locus in human genome by using various approaches. Based on CAGE tags, we found three transcription start regions within a conserved region of 1035 bp upstream of the previously defined STK35 start codon. CAGE is a high-throughput method to measure expression levels by counting large amounts of sequenced capped 5′ ends of transcripts, termed CAGE tags [15], [16]. CAGE tags are an essential resource for profiling transcriptional starting sites and can be used for profiling gene expressions by counting CAGE tags associated with genes. By analyzing the transcription start region TSR_2.030.439–484 we found a start codon AUG surrounded by a Kozak sequence upstream 990 bp of the previously published start codon. The translation initiation sites in eukaryotic mRNAs are reached via a scanning mechanism which predicts that translation should start at the AUG codon nearest the 5′ end of the mRNA [28]. So translation initiation from the previously known start codon which is present in the middle of the second exon is most unlikely. Indeed, this novel open reading frame coded for a protein of 58 kDa, exactly the size of the protein band recognized by the antibody on immunoblot. Moreover, RT-PCR analysis confirmed the presence of the 58 kDa transcript of STK35L1 in endothelial cells. Three orthologous sets of STK35L1 homologs were identified based on syntenic and gene structure pattern data. STK35L1 is conserved in vertebrates. An exception was chicken that had a truncated mRNA starting at the third exon translating only for a partial kinase domain. The present version of the *Gallus* genomic sequence is unable to solve this issue; it can be only clarified with further improvement of genomic assembly of chicken.

*STK35L2* gene is present in all vertebrates in conserved micro-environment without any exceptions. One of the most exciting outcomes of the present analysis is the identification of a third member in this family as *STK35L3*. Its orthologs are found from fish to marsupial mammal. However, this gene is lost in placental mammals after divergence of marsupial and placental mammals at ∼173–190 MY ago. Placental mammals have a conserved syntenic organization, but no *STK35L3* gene. It gives a hint that this gene was lost due to positive selection and might be involved in physiology related to laying eggs or egg development.

The three sets of STK35L1 homologs have independent gene structure patterns, further supporting their orthologous status. Any other known ser/thr kinase family member does not share these patterns of exon-intron organization. An ancestor of the *STK35L1* is identified in two *Ciona* species that are the closest living relatives of vertebrates [29]. Additionally, in our analysis we do not find any evidence to support the presence of *STK35* in sea urchin. Furthermore, no genuine homologs of STK35L1 in worms, flies, echinoderms and lancelets were found even though these groups of invertebrate models possess easily identifiable ser/thr kinases. Thus, it is most likely that the *STK35L1* homologs evolved before the divergence of the vertebrate lineage/*Ciona* lineage about 550 MY ago.

Furthermore, this comprehensive study will provide a platform to further analyze the functional role and regulation of the STK35L1 kinase, and gene transcription in various physiological and patho-physiological conditions.

## Materials and Methods

### Materials

Oligonucleotides and siRNAs were synthesized from MWG Biotech AG (Ebersberg, Germany). Rabbit polyclonal anti-STK35 antibody was generated against the C-terminal peptide 507–521 (DMLANNPQDRPDAF) were generated by Biotrend Chemikalien GmbH (Köln, Germany). Polyclonal goat STK35 antibody was from Everest Biotech (Oxfortshire, UK). Anti-EGFP antibody was kindly gifted by Prof. Schleicher (Institute of Cell Biology, University of Munich, Germany).

### RT–PCR

Total RNA was isolated from Endothelial HeLa and HEK cells, using RNeasy mini kit (Qiagen, Hilden, Germany). First-strand cDNAs were synthesized with Omniscript reverse transcriptase kit (Qiagen, Hilden, Germany) using random hexamer primers as per manufacturer's protocol. cDNAs from these cell types were subjected for gene expression analysis using primers. These primers were designed from different regions of the *STK35L1* gene (Table S1).

### Construction of the expression plasmids

The full-length coding sequence of STK35 and ST35L1 was amplified by PCR from a cDNA pool of human umbilical vein endothelial cell total RNA. The PCR-amplified product of STK35 was cloned into XhoI and HindIII sites of pEGFP-C1 vector (Clontech) to obtain STK35 fused with EGFP. The full length of *STK35L1* gene was obtained using a multistep cloning strategy. In the first step PCR, we amplified 3′ end of *STK35L1* (bp 400–1605) using primers Clik1_long400F and STK35R_HINDIII (Table S1). In second step, the 5′ end (520 bp) of *STK35L1* gene was amplified using 1_F and 520_R. In third step, the PCR fragments from first step and second step were mixed in 1∶3 molar ratios respectively and used as PCR template for amplification of the full length *STK35L1*gene using 1_F and STK35R_HINDIII primers. The deletion mutants of EGFP-STK35 were generated by QuikChange II site-directed mutagenesis kit (Stratagene, La Jolla, CA) as per the manufacturer's instruction. CLP36 gene was amplified as describe previously [5] and cloned into EcoR1 and Sal1 sites of pDsRed-Monomer-N1 (Clontech). All the constructs were confirmed by DNA sequencing (MWG Biotech AG, Ebersberg, Germany).

### Cell culture and transfection

Human umbilical vein endothelial cells were obtained and cultured as described previously [30]. Briefly, cells harvested from umbilical cords were plated onto collagen-coated (room temperature, 75 µg/ml collagen G; Biochrom, Berlin, Germany) plastic culture flasks and were cultured at 5% CO_2_, and 37°C in complete endothelial growth medium (Promo Cell, Heidelberg, Germany). In all experiments, human umbilical vein endothelial cells up to third passage were used. HeLa and HEK cells were grown in OptiMEM medium (Invitrogen) supplemented with 10% fetal calf serum.

Endothelial cells were transfected using the nucleofaction transfection technology developed by Amaxa GmbH as per manufacturer's instructions.

### Confocal microscopy

After 8–10 h of transfection, cells were washed fixed with 3.7% formaldehyde in PBS for 10 minutes at 4°C. For staining of the DNA, cells were incubated with Hoechst 33258 dye solution (1 µg/ml) in PBS for 10 minutes. Cells were observed with a Zeiss LSM510 confocal laser-scanning microscope. The microscope function was controlled by a light manager through the software LSM 510 META.

### Western blot analysis

Transfected or untransfected endothelial cells or nuclear lysates (prepared by using nuclei isolation kit from sigma as per manufacturer's protocol) of HEK, HeLa and endothelial cells were dissolved in an equal volume of 2× Laemmli buffer. Equal amounts of proteins in the samples were subjected to SDS-PAGE and then transferred to nitrocellulose membrane at 200 mAmp for 60 min at 4°C using the Mini Trans-Blot electrophoresis cell (Bio-Rad). Membranes were blocked with 5% (w/v) nonfat milk and incubated with the respective primary and secondary antibodies. The dilutions of the primary antibodies were: anti-STK35 (1∶250) and anti-EGFP (1∶250). The dilution of the secondary antibody (horseradish peroxidase-conjugated) was 1∶5000. The membranes were developed with SuperSignal West Pico chemiluminescent substrate (Pierce) and exposed to Hyperfilm (Amersham Biosciences).

### Data sources of genomic, cDNA, and protein sequences

The genomic DNA/cDNA/protein sequences from different organisms were extracted via different types of BLAST [31] searches using human *STK35L1/PDIK1L* as query sequence from the different genomic databases (Table S2). The newly identified cDNA sequences of *STK35L1* and *STK35L3* are deposited in Genbank. GenBank accession numbers of *STK35L1* and *STK35L3* nucleotide sequences are GQ281297 and GQ281298 respectively. Ensembl accession identifiers of *STK35L1*, *STK35L2* and *STK35L3* genes in different organisms are mention in Table S3.

### Gene structure prediction and mapping introns positions

ElDorado tool from Genomatix GmbH (http://www.genomatix.de/) was used for initial analysis of STK35 gene in human genome. To ensure correct gene structures of all putative novel kinases, gene structures were predicted using GENSCAN [32], [33] and predictions were repeated using GENOMESCAN [32], [33], GENEWISE [34] and FGENESH/FGENESH+ (www.softberry.com). Intron-exon structures were determined with aid of GENEWISE [34] and/or PROT_MAP (www.softberry.com).

### Micro-synteny analysis across different genomes

To verify the orthology, microsynteny across different genomes were analyzed using NCBI mapviewer, ENSEMBL genome browser [35], JGI genome browser (http://www.jgi.doe.gov/), *Tetraodon* genome browser at the Genoscope (http://www.genoscope.cns.fr/externe/tetranew/) and UCSC genome browser [36], [37].

### Sequence alignment of protein kinases

Protein alignments of protein kinases were generated with CLUSTALX 1.83 [38], [39]. The alignments were edited and visualized different sequence characteristics using GENEDOC [40].

### Sequence similarities comparisons between two species

Pairwise protein alignments for *STK35L1* and its exons in two species were carried out by Emboss tool ‘needle’at EBI (http://www.ebi.ac.uk/Tools/emboss/align/index.html).

### Phylogenetic tree analysis

All phylogeny trees were created with full length sequences of representative serine threonine kinases from different organisms with help of Neighbor Join (NJ) method [26] using “MEGA 4”, a molecular evolutionary genetics analysis software [41].

## Supporting Information

Figure S1 Messenger RNA expression of STK35 in various human tissues and cells. The expression profile of STK35 in 79 human tissues is shown here. The expression data was obtained from http://symatlas.gnf.org.
(0.05 MB TIF)

Figure S2 Multiple protein sequence alignment of human STK35L1 homologs from human (Hsap), opossum (Mdom) and its homolog from Ciona (Cint). Human STK35L1 kinase domain has 45.2% identity and 63.7% similarity with Ciona homolog. The position of kinase domain (based on human kinase)is indicated by blue line.
(0.54 MB TIF)

Figure S3 Expression analysis of STK35. Expression analysis of STK35. To verify the expression of STK35 mRNA, RNA and cDNA were prepared from endothelial cells. 455 bp and 755 bp PCR products (Lane 2 and 3) were amplified with STK35 specific primers from cDNA pools. No PCR product was amplified in negative control (reverse transcriptase polymerase was excluded during RT-PCR).
(0.11 MB TIF)

Table S1 List of the primers and their sequences.
(0.04 MB DOC)

Table S2 List of the genomes of selected species investigated in the present study.
(0.04 MB DOC)

Table S3 Ensembl accession identifiers of STK35L1, STK35L2 (PDIK1L), and STK35L3 genes in different organisms. * NCBI accession id. # Species specific paralogs.
(0.12 MB DOC)

Table S4 List of the Markers flanking different STK35L1, PDIK1L, and STK35L3.
(0.04 MB DOC)
