# Supplementary figures and images for: Identifying and Characterizing a Novel Protein Kinase STK35L1 and Deciphering Its Orthologs and Close-Homologs in Vertebrates
Source: PLoS One. 2009 Sep 16;4(9):e6981. doi: 10.1371/journal.pone.0006981 (PMC2737284; doi:10.1371/journal.pone.0006981)

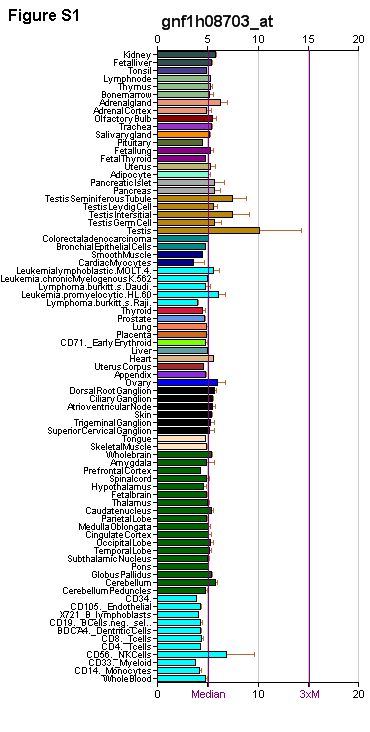

Supplement: Figure S1 — Messenger RNA expression of STK35 in various human tissues and cells. The expression profile of STK35 in 79 human tissues is shown here. The expression data was obtained from http://symatlas.gnf.org. (0.05 MB TIF) [file pone.0006981.s001.tif]

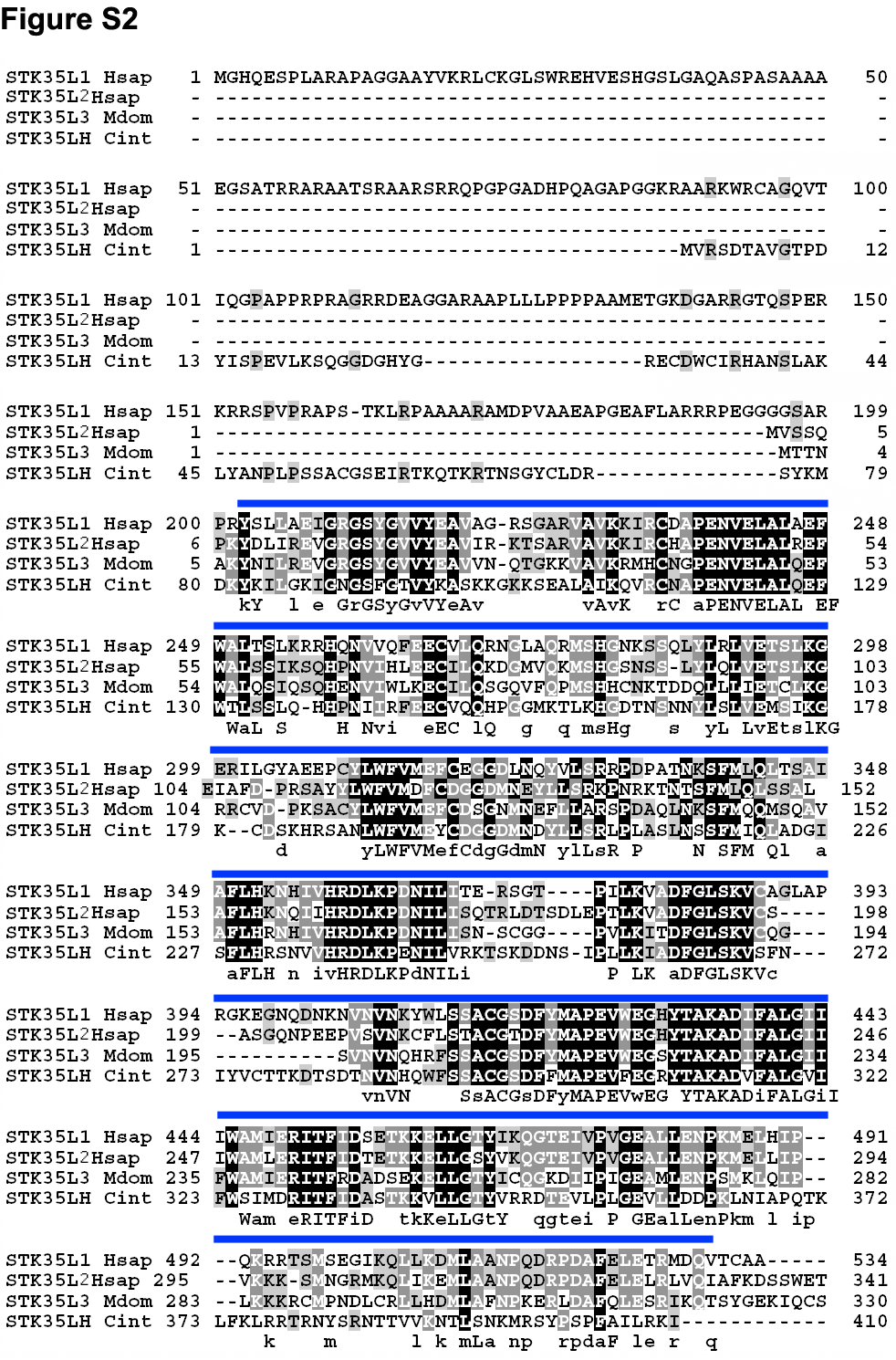

Supplement: Figure S2 — Multiple protein sequence alignment of human STK35L1 homologs from human (Hsap), opossum (Mdom) and its homolog from Ciona (Cint). Human STK35L1 kinase domain has 45.2% identity and 63.7% similarity with Ciona homolog. The position of kinase domain (based on human kinase)is indicated by blue line. (0.54 MB TIF) [file pone.0006981.s002.tif]

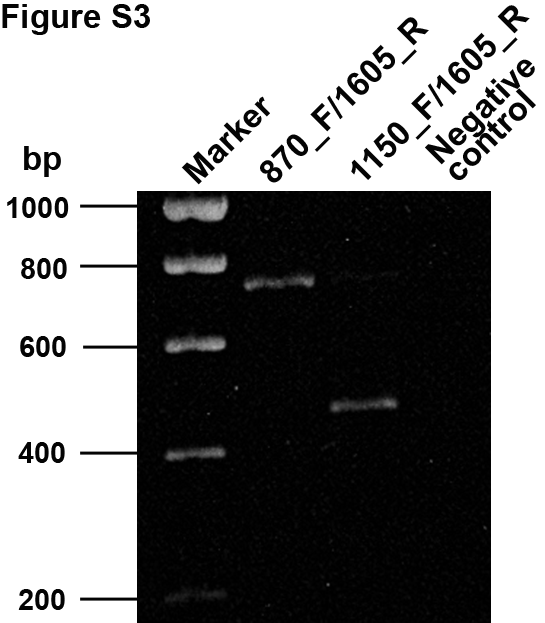

Supplement: Figure S3 — Expression analysis of STK35. Expression analysis of STK35. To verify the expression of STK35 mRNA, RNA and cDNA were prepared from endothelial cells. 455 bp and 755 bp PCR products (Lane 2 and 3) were amplified with STK35 specific primers from cDNA pools. No PCR product was amplified in negative control (reverse transcriptase polymerase was excluded during RT-PCR). (0.11 MB TIF) [file pone.0006981.s003.tif]
