# Supplementary material for: Identifying and Characterizing a Novel Protein Kinase STK35L1 and Deciphering Its Orthologs and Close-Homologs in Vertebrates
Source: PLoS One. 2009 Sep 16;4(9):e6981. doi: 10.1371/journal.pone.0006981 (PMC2737284; doi:10.1371/journal.pone.0006981)
Supplement: Table S1 — List of the primers and their sequences. (0.04 MB DOC) [file pone.0006981.s004.doc]

**Table S1**

| **Primer name** | **Sequence** | | **PCR-Product size** |
| --- | --- | --- | --- |
| Beta actin forw_258 | CAC CAC ACC TTC TAC AAT GAG C | | 179 bp |
| Beta actin rev_437 | CAG AGG CGT ACA GGG ATA GC | |
| STK35_RTPCR_F_471 | ggt gga gac ctc gct gaa ag | | 273 bp |
| STK35_RT_R_744 | CTA AGC AAG GTC TGT GCT GG | |
| 1_F | Atg ggc cac cag gag tct ccg ctg g | |  |
| 520_R | GGGCCTCGGCCGCCACCGGATC | |  |
| 197_F | CTCGGTCCCGGAGGCAGCCCGG | |  |
| 870_F | CC ggt gga gac ctc gct gaa ag | |  |
| 1143_R | gtcggccactttgaggatgg | |  |
| STK35_F_XHO1 | ATTActcgagctATGGAAACGG GGAAG | |  |
| STK35_R_HINDIII | ATTAAAGCTTAAGCAGCA CATGTG | |  |
| STK35L_1_Xho_F | CAGATCTCGAGCTATGGGCCACCAGGAGTCTCCGC | |  |
| Clik1_long400F | ATG GAA ACG GGG AAG GAC GGC GCC CGC | |  |
| **SiRNA** | **Sequence** | **Distance from Start codon in bp** | |
| siSTK35L1_1111 | GCU CUU GAA AGA UAU GUU AGC | 1512 | |
| siSTK35L1_5816 | GAC CUA CGU GUG GUG AAC UTT | 6215 | |
| siSTK35L1_829 | AAU GUG AAU GUG AAU AAG UAC | 1238 | |
