# Supplementary material for: Identifying and Characterizing a Novel Protein Kinase STK35L1 and Deciphering Its Orthologs and Close-Homologs in Vertebrates
Source: PLoS One. 2009 Sep 16;4(9):e6981. doi: 10.1371/journal.pone.0006981 (PMC2737284; doi:10.1371/journal.pone.0006981)
Supplement: Table S2 — List of the genomes of selected species investigated in the present study. (0.04 MB DOC) [file pone.0006981.s005.doc]

**Table S2**

| **Name of Genome** | **Version Assembly** | **Website** |
| --- | --- | --- |
| *Homo sapiens* | *NCBI Build 36.2 (Sept.,2006)* | *http://www.ncbi.nlm.nih.gov/projects/mapview/map_search.cgi?taxid=9606* |
|  |  |  |
| ***Xenopus tropicalis*** | V.4.1 (Aug., 2005) | *http://genome.jgi-psf.org/Xentr4/Xentr4.home.html* |
|  |  |  |
| Gallus gallus | WASHUC2.1 (May 2006) | *http://www.ncbi.nlm.nih.gov/projects/mapview/map_search.cgi?taxid=9031* |
|  |  |  |
| Fugu rubripes | V.4.0 (Oct., 2004) | *http://genome.jgi-psf.org/Takru4/Takru4.home.html* |
|  |  |  |
| *Danio rerio* | Zv6 (March 2006) | *http://www.ncbi.nlm.nih.gov/projects/mapview/map_search.cgi?taxid=7955* |
|  |  |  |
| *Tetraodon nigroviridis* | V7 (April 2003) | *http://www.genoscope.cns.fr/externe/tetranew/* |
|  |  |  |
| *Branchiostoma floridea* | V1 (March 2006) | *http://genome.jgi-psf.org/Brafl1/Brafl1.home.html* |
|  |  |  |
| *Ciona intestinalis* | V2.0 (March 2005) | *http://genome.jgi-psf.org/Cioin2/Cioin2.home.html* |
|  |  |  |
| *Strongylocentrotus purpuratus* | Spur_2.1 (Sept., 2006) | *http://www.hgsc.bcm.tmc.edu/projects/seaurchin/* |
|  |  |  |
| *Drosophila melanogaster* | *Release 5* | *http://www.fruitfly.org/* |
|  |  |  |
| *Caenorhabditis elegans* | *Release WS187 (Feb. 2008)* | *http://www.wormbase.org/* |
