# Supplementary material for: Identifying and Characterizing a Novel Protein Kinase STK35L1 and Deciphering Its Orthologs and Close-Homologs in Vertebrates
Source: PLoS One. 2009 Sep 16;4(9):e6981. doi: 10.1371/journal.pone.0006981 (PMC2737284; doi:10.1371/journal.pone.0006981)
Supplement: Table S3 — Ensembl accession identifiers of STK35L1, STK35L2 (PDIK1L), and STK35L3 genes in different organisms. * NCBI accession id. # Species specific paralogs. (0.12 MB DOC) [file pone.0006981.s006.doc]

**Table S3.**

|  |  |  |  |
| --- | --- | --- | --- |
| 1. **STK35L1** | | |  |
| **Organism** | **Species** | **Abbr.** | **Accession id** |
| Human | *Homo sapiens* | Hsap | ENSP00000370891 |
| Mouse | *Mus musculus* | Mmus | ENSMUSP00000080721 |
| Chimpanzee | *Pan troglodytes* | Ptro | ENSPTRP00000022552 |
| Rat | *Rattus norvegicus* | Rnor | ENSRNOP00000008071 |
| Elephant | *Loxodonta africana* | Lafr | ENSLAFP00000003180 |
| Hyrax | *Procavia capensis* | Pcap | ENSPCAP00000006647 |
| Lesser hedgehog tenrec | *Echinops telfairi* | Etel | ENSETEP00000004371 |
| Dolphin | *Tursiops truncates* | Ttru | ENSTTRP00000010224 |
| Cow | *Bos taurus* | Btau | ENSBTAP00000004632 |
| Hedgehog | *Erinaceus europaeus* | Eeur | ENSEEUP00000004179 |
| Shrew | *Sorex araneus* | Sara | ENSSARP00000003728 |
| Megabat | *Pteropus vampyrus* | Pvam | ENSPVAP00000011656 |
| Microbat | *Myotis lucifugus* | Mluc | ENSMLUP00000000689 |
| Dog | *Canis familiaris* | Cfam | ENSCAFP00000010065 |
| Gorilla | *Gorilla gorilla* | Ggor | ENSGGOP00000004754 |
| Orangutan | *Pongo pygmaeus* | Ppyg | ENSPPYP00000012110 |
| Macaque | *Macaca mulatta* | Mmul | ENSMMUP00000005759 |
| Tarsier | *Tarsius syrichta* | Tsyr | ENSTSYP00000005920 |
| Microbat | *Microcebus murinus* | Mmur | ENSMICP00000013145 |
| Bushbaby | *Otolemur garnettii* | Ogar | ENSOGAP00000015275 |
| Alpaca | *Vicugna pacos* | Vpac | ENSVPAP00000001119 |
| Horse | *Equus caballus* | Ecab | ENSECAP00000000144 |
| Kangaroo rat | *Dipodomys ordii* | Dord | ENSDORP00000006337 |
| Guinea Pig | *Cavia porcellus* | Cpor | ENSCPOP00000002034 |
| Rabbit | *Oryctolagus cuniculus* | Ocun | ENSOCUP00000000784 |
| Pika | *Ochotona princeps* | Opri | ENSOPRP00000006783 |
| Northern treeshrew | *Tupaia belangeri* | Tbel | ENSTBEP00000015031 |
| Armadillo | *Dasypus novemcinctus* | Dnov | ENSDNOP00000010346 |
| Opossum | *Monodelphis domestica* | Mdom | ENSMODP00000017441 |
| Chicken | *Gallus gallus* | Ggal | BU403355^*^ |
| Frog | *Xenopus tropicalis* | Xtro | ENSXETP00000042647 |
| Pufferfish (Fugu) | *Takifugu rubripes* | Trub | ENSTRUP00000030700 |
| Pufferfish (Fugu) | *Takifugu rubripes* | Trub | ENSTRUP00000023999^#^ |
| Green spotted pufferfish | *Tetraodon nigroviridis* | Tnig | ENSTNIP00000004584 |
| Green spotted pufferfish | *Tetraodon nigroviridis* | Tnig | ENSTNIP00000005692^#^ |
| Zebrafish | *Danio rerio* | Drer | ENSDARP00000093577 |
| Zebrafish | *Danio rerio* | Drer | ENSDARP00000093600^#^ |
| Zebrafish | *Danio rerio* | Drer | ENSDARP00000053186^#^ |
| Pufferfish (Fugu) | *Takifugu rubripes* | Trub | ENSTRUP00000023999^#^ |
| Sicklefish | *Gasterosteus aculeatus* | Gacu | ENSGACP00000004732 |
| Sicklefish | *Gasterosteus aculeatus* | Gacu | ENSGACP00000015240^#^ |
| Medaka | *Oryzias latipes* | Olat | ENSORLP00000023463 |
|  |  |  |  |
| 1. **STK35L2 (PDIK1L)** | | |  |
| **Organism** | **Species** | **Abbr.** | **Accession id** |
| Human | *Homo sapiens* | Hsap | ENSP00000363389 |
| Mouse | *Mus musculus* | Mmus | ENSMUSP00000101503 |
| Rat | *Rattus norvegicus* | Rnor | ENSRNOP00000022141 |
| Chimpanzee | *Pan troglodytes* | Ptro | ENSPTRP00000000659 |
| Orangutan | *Pongo pygmaeus* | Ppyg | ENSPPYP00000001958 |
| Macaque | *Macaca mulatta* | Mmul | ENSMMUP00000022182 |
| Tarsier | *Tarsius syrichta* | Tsyr | ENSTSYP00000009722 |
| Gray Mouse Lemur | *Microcebus murinus* | Mmur | ENSMICP00000015913 |
| Bushbaby | *Otolemur garnettii* | Ogar | ENSOGAP00000000519 |
| Squirrels | *Spermophilus tridecemlineatus* | Stri | ENSSTOP00000004470 |
| Kangaroo rat | *Dipodomys ordii* | Dord | ENSDORP00000003988 |
| Guinea Pig | *Cavia porcellus* | Cpor | ENSCPOP00000010516 |
| Rabbit | *Oryctolagus cuniculus* | Ocun | ENSOCUP00000009426 |
| Pika | *Ochotona princeps* | Opri | ENSOPRP00000001295 |
| Northern treeshrew | *Tupaia belangeri* | Tbel | ENSTBEP00000002302 |
| Dolphin | *Tursiops truncates* | Ttru | ENSTTRP00000004832 |
| Cow | *Bos taurus* | Btau | ENSBTAP00000001982 |
| Alpaca | *Vicugna pacos* | Vpac | ENSVPAP00000001135 |
| Guinea Pig | *Cavia porcellus* | Cpor | ENSCPOP00000017067 |
| Shrew | *Sorex araneus* | Sara | ENSSARP00000009146 |
| Hedgehog | *Erinaceus europaeus* | Eeur | ENSEEUP00000006394 |
| Megabat | *Pteropus vampyrus* | Pvam | ENSPVAP00000006004 |
| Microbat | *Myotis lucifugus* | Mluc | ENSMLUP00000014266 |
| Dog | *Canis familiaris* | Cfam | ENSCAFP00000018629 |
| Horse | *Equus caballus* | Ecab | ENSECAP00000021911 |
| Elephant | *Loxodonta africana* | Lafr | ENSLAFP00000002042 |
| Hyrax | *Echinops telfairi* | Etel | ENSETEP00000003044 |
| Opossum | *Monodelphis domestica* | Mdom | ENSMODP00000017631 |
| Platypus | *Ornithorhynchus anatinus* | Oana | ENSOANP00000023451 |
| Chicken | *Gallus gallus* | Ggal | ENSGALP00000002216 |
| Frog | *Xenopus tropicalis* | Xtro | ENSXETP00000020181 |
| Pufferfish (Fugu) | *Takifugu rubripes* | Trub | ENSTRUP00000023869 |
| Green spotted pufferfish | *Tetraodon nigroviridis* | Tnig | ENSTNIP00000012691 |
| Sicklefish | *Gasterosteus aculeatus* | Gacu | ENSGACP00000010368 |
| Medaka | *Oryzias latipes* | Olat | ENSORLP00000010301 |
| Zebrafish | *Danio rerio* | Drer | ENSDARP00000073471 |
|  |  |  |  |
| 1. **STK35L3** | | | |
| **Organism** | **Species** | **Abbr.** | **Accession id** |
| Opossum | *Monodelphis domestica* | Mdom | ENSMODP00000010759 |
| Opossum | *Monodelphis domestica* | Mdom | ENSMODP00000026975^#^ |
| Chicken | *Gallus gallus* | Ggal | AJ720485.1^*^ |
| Frog | *Xenopus tropicalis* | Xtro | ENSXETP00000045741 |
| Green spotted Pufferfish | *Tetraodon nigroviridis* | Tnig | ENSTNIP00000022367 |
| Pufferfish (Fugu) | *Takifugu rubripes* | Trub | ENSTRUP00000005966 |
| Medaka | *Oryzias latipes* | Olat | ENSORLP00000020278 |
| Sicklefish | *Gasterosteus aculeatus* | Gacu | ENSGACP00000005871 |
| Zebrafish | *Danio rerio* | Drer | ENSDARP00000061554 |
|  |  |  |  |
| 1. **Ancestor of STK35L1 gene from sea squirt genomes** | | |  |
| **Organism** | **Species** | **Abbr.** | **Accession id** |
| Sea squirt | *Ciona intestinalis* | Cint | ENSCINP00000006268 |
| Sea squirt | *Ciona savigyni* | Csav | ENSCSAVP00000013846 |
|  |  |  |  |

* NCBI accession id.

# Species specific paralogs.
