# Supplementary material for: Identifying and Characterizing a Novel Protein Kinase STK35L1 and Deciphering Its Orthologs and Close-Homologs in Vertebrates
Source: PLoS One. 2009 Sep 16;4(9):e6981. doi: 10.1371/journal.pone.0006981 (PMC2737284; doi:10.1371/journal.pone.0006981)
Supplement: Table S4 — List of the Markers flanking different STK35L1, PDIK1L, and STK35L3. (0.04 MB DOC) [file pone.0006981.s007.doc]

**Table S4.**

| **Markers flanking STK35L1** | |
| --- | --- |
| PYDN | Prodynorphin |
| STK38 | Serine/threonine kinase 38 |
| **Markers flanking STK35L2** | |
| SF3A3 | Splicing factor 3A subunit 3 (Spliceosome-associated protein 61) |
| MANEAL | Glycoprotein endo-alpha-1,2-mannosidase-like protein |
| CITED4 | Cbp/p300-interacting transactivator 4 (MSG1-related protein 2)(MRG-2) |
| LIN28 | Lin-28 homolog A (Zinc finger CCHC domain-containing protein 1) |
| STMN1 | Stathmin (Phosphoprotein p19) |
| EXTL1 | Exostoses |
| PAFAH2 | Platelet-activating factor acetylhydrolase 2 Gene |
| TRIM63 | Tripartite motif-containing protein 63 |
| **Markers flanking STK35L3** | |
| HMBOX1 | Homeobox-containing protein 1 |
| ELP3 | Elongator complex protein 3 (hELP3) |
| EMILIN1 | EMILIN-1 Precursor (Elastin microfibril interface-located protein 1 |
| ANKRD5 | Ankyrin repeat domain-containing protein 5 |
| PNOC | Nociceptin Precursor [Contains Neuropeptide 1;Nociceptin(Orphanin FQ)(PPNOC);Neuropeptide 2] |
